# Informing the redesign of psychiatric seclusion rooms: a mixed-methods pre-evaluation with individuals with lived experience

**DOI:** 10.1186/s12888-026-07780-0

**Published:** 2026-01-16

**Authors:** Leonie Ascone, Candelaria Mahlke, Nour Tawil, Larissa Samaan, Martin Frisch, Lena Nugent, Rebecca Nixdorf, Florian Börncke, Daniel Lüdecke, Rabea Fischer, Nora Bach, Cindy Hackbarth, Timothy McCall, Jürgen Gallinat, Simone Kühn

**Affiliations:** 1 Neuroplasticity Research Group, Department of Psychiatry and Psychotherapy, Center for Psychosocial Medicine, University Medical Center Hamburg-Eppendorf, Martinistraße 52, 20246 Hamburg, Germany; 2 Participatory and Collaborative Research Group, Department of Psychiatry and Psychotherapy, Center for Psychosocial Medicine, University Medical Center Hamburg-Eppendorf, Martinistraße 52, 20246 Hamburg, Germany; 3 Center for Environmental Neuroscience, Max Planck Institute for Human Development, Lentzeallee 94, 14195 Berlin, Germany; 4 Station PAEG (Acute Psychiatric Unit), Department of Psychiatry and Psychotherapy, Center for Psychosocial Medicine, University Medical Center Hamburg-Eppendorf, Martinistraße 52, 20246 Hamburg, Germany; 5 Station PA2 (Psychosis Treatment Unit), Department of Psychiatry and Psychotherapy, Center for Psychosocial Medicine, University Medical Center Hamburg-Eppendorf, Martinistraße 52, 20246 Hamburg, Germany; 6 Sustainable Environmental Health Sciences, Medical School OWL, Bielefeld University, Universitätsstraße 25, 33615 Bielefeld, Germany

**Keywords:** Acute psychiatry, Coercion, Lived experience, Wall design, Biophilia, Qualitative research

## Abstract

**Background:**

In acute psychiatric inpatient settings, where perception is altered and emotional vulnerability is heightened, many facilities use coercive seclusion rooms for safety. This practice has been increasingly criticized for its psychological impact. While architectural guidelines emphasize human-centered and biophilic design to support well-being, empirical evidence on how design features in this specific setting affect patients remains limited.

**Methods:**

This mixed-methods study examined how individuals with lived experience of coercive isolation in seclusion rooms (*N* = 30) perceived different wall designs. The participants viewed ten digitally rendered images of a to-be-redesigned seclusion room, including furniture, varying in wall design (six nature-themed images, three wall colors: blue, green, beige; and a white empty control room) and rated each on restfulness, stress, liking, and overwhelm. This was followed by qualitative interviews on needs and design preferences.

**Results:**

Nature-themed wallpapers, especially an image of grass-covered dunes by the sea, and blue and green wall colors were rated as more restorative and less stressful than both the empty white control room and a beige-painted comparison room, or a more complex, ‘rough’ wilderness nature image. Qualitative findings emphasized calm, homelike, nature-themed, and controllable environments, orientation, more transparent and empathetic communication, and respectful care.

**Conclusions:**

Individuals with lived experience respond more favorably to blue and green color schemes and non-complex, calm nature imagery, compared to more neutral or sterile low-stimulation designs, challenging long-standing assumptions that sensory deprivation best supports de-escalation in seclusion. The study further illustrates the feasibility and ethical value of participatory pre-evaluative research to inform design.

**Trial registration:**

The study and main analyses were pre-registered 2024/08/22 at aspredicted.org (#187275) https://aspredicted.org/9ht9-ckwn.pdf.

**Clinical trial number:**

Not applicable.

**Supplementary Information:**

The online version contains supplementary material available at 10.1186/s12888-026-07780-0.

## Background

Acute psychiatric environments face a fundamental design challenge: spaces built to ensure containment, safety, and efficiency often compromise psychological well-being. While these priorities are essential, the resulting environments can elevate fear, stress, and loss of control. In recent decades, the field of *healing architecture* [1] has emphasized that the built environment can and should actively support recovery. This approach draws on psychological theories highlighting the role of nature in human well-being, such as Ulrich’s *Stress Recovery Theory* (SRT) [2], Kaplan’s *Attention Restoration Theory* (ART) [3], and the *biophilia hypothesis* [4]. All converge on the notion that exposure to nature or nature-related elements supports well-being; whether through emotional regulation and stress recovery (SRT) or cognitive restoration (ART) elicited by nature’s unique role for human thriving. Healing architecture therefore integrates natural elements, materials, and design principles, a concept termed *biophilic design* [5]. Architecture is increasingly understood as a complex intervention that must balance utility, efficiency, and safety with a social-dynamic, human-centered, interactional (environment-person fit) and salutogenic perspective. Despite growing implementation of these ideas in healthcare design, targeted research on which specific design elements (e.g., colors, materials, or imagery) effectively convey mental health effects or other desirable outcomes remains scarce.

Our research group initiated a multi-phase therapeutic and environmental redesign program of our closed psychiatric acute ward (*PAEG.change*), initially focusing on the redesign of two isolation/ seclusion rooms (*ISO.change project*; AsPredicted #175723). Planned features include a media wall for safe activities, adaptive RGB lighting, improved climate control, window views of sky/ nature, safe furniture, and the use of wall color and/or natural imagery. Pre-post changes in patient outcomes, both self-reported and by staff-observation, will be evaluated. The present preregistered pilot experiment (*E-Wall-Uation*; AsPredicted #9ht9-ckwn) represented both an independent study and a preparatory step in this process. Specifically, we focused on wall design, one of the most visually dominant and easily modifiable room components. The aims were (a) to identify (visually) calming and non-triggering wall design options and (b) to gather qualitative input on patient needs and preferences for seclusion spaces within acute psychiatric settings, including their potential voluntary use as retreat rooms. The study population were individuals with lived experience. In the following we summarize the theoretical and empirical evidence which informed our room concept and methodological approach.

### Design guidelines and evidence of design effects of psychiatric inpatient and acute care facilities

International guidelines, such as from the U.S. Department of Veteran Affairs [6], the UK NHS [7], the National Association for Psychiatric Intensive Care Units (NAPICU) [8], or the German *Planungshilfe Deeskalierendes Bauen* (Lower Saxony Ministry) [9], stress that psychiatric emergency healthcare environments need to balance safety and surveillance with dignity, autonomy, sensory control, social interaction vs. retreat possibilities, and calm. Common recommendations include single rooms, designated social interaction and therapeutic spaces, retreat areas, daylight and natural views, warm non-institutional materials, homelike furnishing, and soothing colors. Coercive isolation in seclusion rooms is viewed critically. Beyond considering sanitary (separate areas), hygiene (easy to clean materials/ surfaces), social (constant, 1:1 contact with personnel), and safety (suicide/ self-harm prevention) issues, design recommendations include: lighting, nature views, a calming, home-like atmosphere, safe yet cozy furniture, environmental control possibilities for patients, media (e.g., music, acoustic stimulation), and orientation (time and day). Such rooms are also recommended to be used as voluntary retreat space for self-de-escalation and de-stigmatization [9]. Architecture is increasingly understood as part of the therapeutic process. Empirical evaluations of new facilities built based upon these principles show reductions in coercive measures and improvements in (perceived) safety [10, 11]. However, these complex interventions and related studies rarely isolate specific rooms or design components that may drive such effects.

### Effects of color in interior (healthcare) design

Systematic research on color in (acute) psychiatry is largely absent. Guidelines for healthcare design broadly recommend soothing, non-institutional palettes rather than prescribing specific hues [6, 12]. Experimental research in existing or virtual indoor environments [13–16] has been mostly conducted in students, showing that cool, low-saturation wall colors, especially blue and green, are perceived as calming and restorative with related physiological effects, while warm or saturated tones (e.g., red, yellow) evoke higher arousal. Within healthcare, similar trends appear: for instance, blue and green (vs. yellow or white) walls in patient rooms descriptively improved self-reported mood and stress in mobility-impaired patients [17], and surveys of design experts most strongly link these hues with relaxation, cleanliness, and safety [18]. Tofle et al. [12] concluded that while empirical data are limited, consensus favors blue and green for restorative areas in healthcare settings. To our knowledge, no studies have specifically examined patient responses to wall colors in psychiatric facilities, including seclusion rooms.

#### Nature imagery in healthcare and psychiatric settings

A systematic review of 37 indoor-context studies, mostly conducted in healthy participants, found that exposure to nature images or videos consistently reduced heart rate, blood pressure, skin conductance levels, and prefrontal cortex activation [19]. The health benefits of art and nature imagery on walls in healthcare environments were summarized in a recent critical review [20]. Across 25 experimental studies, most research focused on waiting areas and procedure rooms, and fewer studies examined patient rooms. The review found that photorealistic nature imagery yielded relatively robust and consistent reductions in stress, pain, and anxiety, although the evidence for patient rooms was less conclusive. Only one empirical study in a psychiatric setting was identified [21], showing that a photographic image of nature, but not painted nature or abstract art, was associated with reduced use of as-needed (PRN) medication in an acute care unit’s multifunctional room. In the absence of further evidence and based on clinical considerations of highly stressed acute inpatients, photorealistic, low-saturation, and low-complexity nature imagery hence appears to be most promising.

### Curved vs. angular forms in interior design

**Fig. 1:**
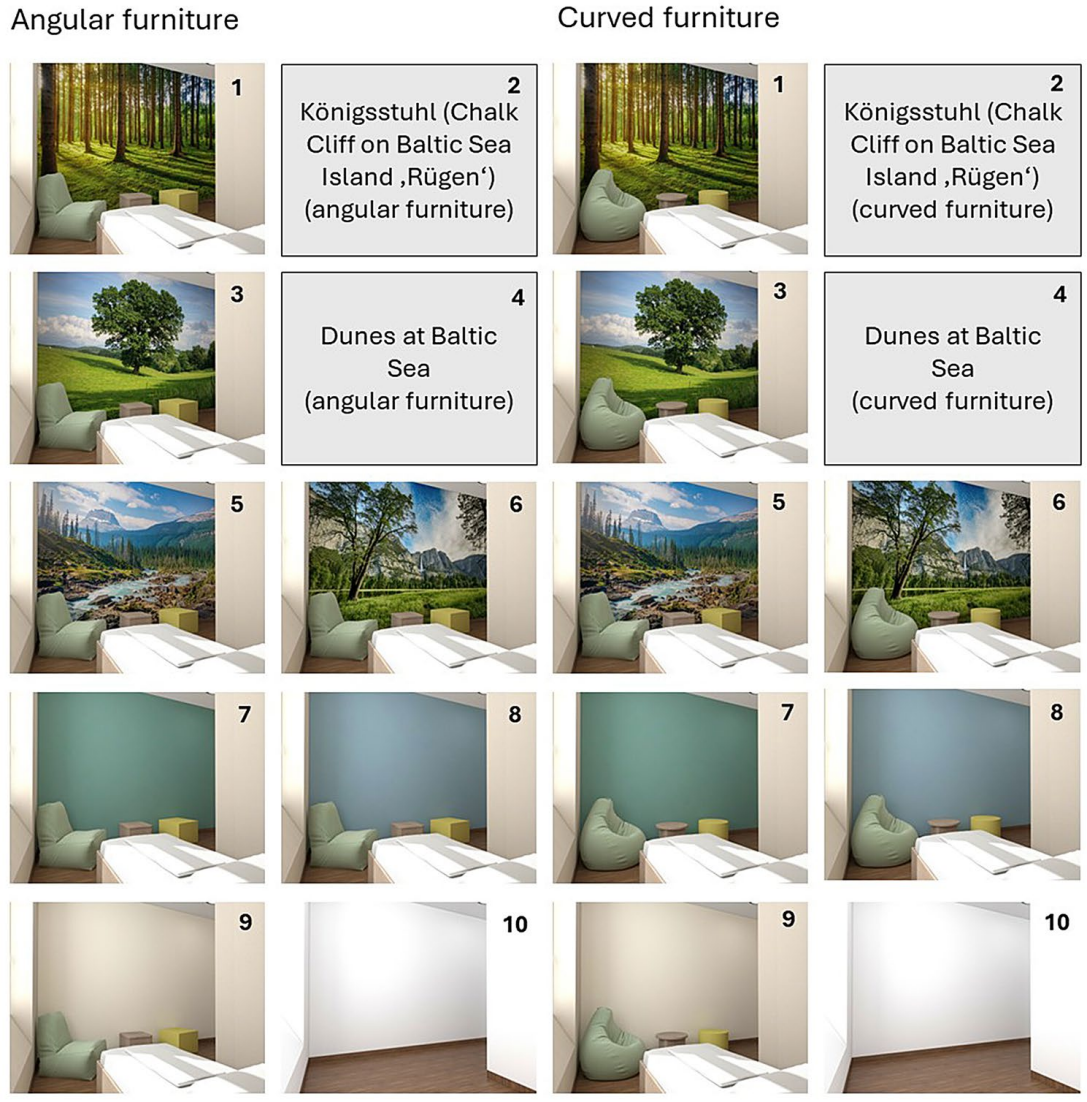
Wall design stimuli used in the seclusion-room experiment. Note. Due to copyright restrictions, the images shown as grey placeholders cannot be displayed here. Links and source details for all six natural landscapes are provided in the Supplementary Materials (Excel file) for this paper. The six nature-based wall designs were labelled: (1) Pine Tree Forest, (2) Königsstuhl (Chalk Cliff on the Baltic Sea Island ‘Rügen’), (3) Oak Tree on a Meadow, (4) Dunes at Baltic Sea, (5) River Running Through Wilderness, and (6) Grass Field Near Mountain With Waterfall. Each design (except the empty control room) was shown with either angular or curved furniture, yielding ten total stimuli per participant

A recent meta-analysis of 61 studies found a general human preference for curved over angular shapes, particularly for everyday objects, and most pronounced based on explicit questions or when requesting quick judgments [22]. Research in healthy populations suggests that curved interior forms, including furniture, may evoke greater comfort and relaxation [23]. In mental health settings, where both safety and sensory calm are crucial, it appears advisable to minimize edges and use round shapes. The present study therefore included a subtle, exploratory manipulation of (angular vs. curved) furniture shape (see Fig. 1).

### Lived experience of seclusion in acute psychiatric care and participatory design

Qualitative research consistently shows that seclusion is experienced as frightening, isolating, and depersonalizing, though occasionally also as protective. A meta-ethnographic synthesis identified recurring themes of trauma, powerlessness, lack of transparency, and need for more dignity, autonomy and control [24]. Participatory design that integrates lived experience can translate these insights into humane environments. For example, Faerden et al. [25] implemented a user-driven redesign of an acute psychiatric seclusion unit to enhance dignity, compared to a non-refurbished control ward. The intervention emphasized a homelike and nature-oriented atmosphere, greater privacy and self-control, improved spatial orientation and staff contact, and realistic consideration of patient needs. Staff ratings before and after the redesign indicated meaningful improvements in perceived environmental support for both patients and staff.

### The present study

This exploratory mixed-method pilot study aimed to inform the redesign of our psychiatric acute ward’s seclusion rooms toward a more recovery-oriented model. Two major research questions were addressed:

1. Quantitative: Which wall designs (diverse nature imagery, colored walls vs. white control room condition) are associated with the most favorable ratings of restfulness, stress, liking, overwhelm and overall preference in a ranking-task? Subtle variations in furniture form were also explored as a secondary factor.
2. Qualitative: What needs and preferences do participants openly describe regarding room design and care during psychiatric crises?

## Methods

The study was approved by the local psychological ethics committee (vote ID: LPEK-0560, 20 Nov 2022). The procedure and materials were reviewed with EmPeeRie, a local scientific advisory board of individuals with lived experience. Six eligibility criteria for study participants were applied: (1) having experienced being isolated in a seclusion room at least once and being able to recall it, (2) at least 18 years old, (3) sufficient stability for study participation, (4) no severe formal thought disorders, mania, or strong negative symptoms, (5) normal or corrected vision and no color blindness, (6) ability to provide informed consent. All participants were informed about the broader aim of improving the situation in the seclusion rooms of our psychiatric acute ward. The researchers, who were not part of the treatment team, first obtained informed consent and collected sociodemographic data. Diagnoses and seclusion histories (frequency, timing, location) were retrieved from casefiles and/or self-reports. In randomized order, participants then viewed nine digitally rendered rooms with specific wall designs (six nature wallpapers, three wall colors [blue, green, beige; see Room design stimuli section for details]) and one empty control room (psychiatric paradigm of ‘sensory deprivation’). Images were projected onto a white wall, with a projection size of approximately 134 × 75 inches (340 × 190 cm). A Sharp PG-D2870W projector (3000 lumens, 1280 × 800 resolution) was used. For each image, participants were asked to remember being in a mental crisis, imagine being in the presented room, and provide ratings on restfulness, stress, liking, and overwhelm in reference to the room (each rated on a 0–6 Likert scale; for details, see Ratings of room designs section).

After the rating session, participants additionally completed a deck sorting task by ranking printed images of all ten rooms from most to least preferred. Finally, participants responded to open-ended questions about their ideal crisis or retreat room: its (design) features, personal needs (what they would wish for), stressors, and what should be avoided. They were also encouraged to reflect freely on the concept of isolation rooms per se, including abolishing them altogether. All responses were noted and verified for accuracy. The (translated from German) questionnaire items can be found in the Supplementary Materials.

### Room design stimuli

Out of ten total room designs (for an overview, see Fig. 1), six included wallpapers with natural landscapes without any humans, built elements, or large, visible animals: (1) Pine Tree Forest, (2) Königsstuhl (Chalk Cliff on the Baltic Sea Island ‘Rügen’), (3) Oak Tree on a Meadow, (4) Dunes at Baltic Sea, (5) River Running Through Wilderness, and (6) Grass Field near Mountain With Waterfall. Images were commercially available wallpapers or high-resolution printable images to be able to later implement the preferred motifs on the walls of the isolation rooms at our own psychiatric ward. Landscape image selection was based on a small-scale pre-study (see Supplementary Materials for details). Twenty high-resolution nature images were evaluated by a panel of seven colleagues with backgrounds in architecture, landscape, environmental neurosciences/ psychology, or psychiatry, on affective and theoretical landscape dimensions. Based on standardized rankings, six top-rated images, also diverse in landscape type and water content, were selected. Furthermore, three colored rooms were presented: with green, blue, and beige wall design (see Table 1 for color specifications). A white, empty version of the room was created, reflecting the psychiatric paradigm of ‘sensory deprivation’. All rooms except the ‘control room’ were additionally furnished – either (as per random group assignment of participants) with a curved vs. angular seating arrangement (curved vs. angular bean bags, coffee tables, and a seating cylinder vs. cube) in front of the designed wall. The furniture was inspired by commercially available products designed specifically for (high-risk) psychiatric settings (Pineapple Contracts Ltd., UK). Initial suggestions for wall images and colors, as well as furniture, had been discussed with the scientific advisory board of individuals with lived experience in an advisory session about the planned study and planned redesign (still ongoing) within our own acute ward. Within the redesign and research process, we are also constantly involving a peer worker with lived experience.

**Table 1:** Color specifications for the colored walls

**Color**			
	Blue	Green	Beige
**HEX**	#89A9B7	#709C93	#F9F2E2
**RGB**	137, 169, 183	112, 156, 147	249, 242, 226

### Ratings of room designs

The selection of the four rating dimensions (restfulness, stress, liking, and overwhelm) was guided by theoretical and empirical considerations. Restfulness and stress reflect core constructs of SRT [2] and ART [3]. Specifically, SRT emphasizes the immediate affective and physiological benefits of exposure to calming, non-threatening natural settings, such as reductions in arousal, tension, and negative mood, while ART highlights the cognitive benefits of environments rich in “soft fascination” (in stark contrast to complex, chaotic, or even threatening urban scenes), allowing directed attention to rest and thereby improving concentration, problem-solving, and self-regulation capacities. Liking was included as a measure of aesthetic preference, which is theoretically relevant for environments intended to support dignity and comfort. The fourth dimension, disturbance (or overwhelm), was incorporated to capture potential adverse responses to sensory load, based on pilot feedback from both clinicians and the scientific advisory board of individuals with lived experience. To reduce cognitive burden and ensure accessibility for participants in vulnerable psychological states, we used single-item ratings for each construct. The items were co-designed with the local scientific advisory board of individuals with lived psychiatric experience from our clinic. This process included the refinement of wording to assure they were understood by patients and developing a color-coded 6-point Likert scale (ranging from red = highly negative to green = highly positive).

Similar single-item measures of restfulness, stress, and liking have been used in prior experimental studies in architectural and environmental psychology, demonstrating acceptable face validity and relevance for spatial and affective evaluation [23, 26]. Because direct comparisons to previous work are limited due to differences in wording and scale format, we calculated inter-item correlations across all room designs to explore internal coherence. These analyses revealed moderate to strong significant, theory-consistent associations (see Table 2), supporting the construct validity of the items.

**Table 2:** Pearson correlation ranges between rating dimensions across rated room designs

	MIN	MAX
Restfulness – liking	0.700	0.942
Restfulness – stress	− 0.456	− 0.903
Restfulness - disturbance	− 0.424	− 0.875
Liking – stress	− 0.577	− 0.892
Liking – disturbance	− 0.513	− 0.839
Stress – disturbance	0.567	0.916

Note. All correlations were significant at least at the *p* < .05 level

#### Statistical analyses

The study and main analyses were pre-registered (2024/08/22 at aspredicted.org (#187275) https://aspredicted.org/9ht9-ckwn.pdf). The exploratory research question was whether patient ratings of restfulness, stress, liking, and overwhelm differed significantly across wall designs and form. A factorial design was employed with furniture form (angular vs. curved) as randomly assigned between-subjects factor and wall design as within-subjects factor. The main effect of wall design was the main focus, but also main effects of form and the interaction between wall design and form were analyzed using repeated-measures mixed ANOVA in SPSS 27. Post-hoc tests (in case of significant main effects or interactions) to identify best-rated images were run using Wilcoxon signed rank tests, with resulting *p*-levels being adjusted for multiple testing using the Benjamini-Hochberg false discovery rate (FDR) correction [27], applied separately within each rating dimension (restfulness, stress, liking, overwhelm).

#### Qualitative analyses

To address the research question “*How should seclusion/ isolation rooms be designed to adequately support the needs of users?*”, qualitative data from the open-ended questions (for details see Supplementary Materials) were analyzed. Participants were asked to describe their ideal crisis or retreat room design and treatment-wise preferences. The term “seclusion” or “isolation room” was deliberately avoided to stimulate participants’ imagination considering alternative models where rooms serve as voluntary retreat spaces rather than for coercive confinement. Data were analyzed by NB in a first round and refined by CM in a second round, using inductive category formation following Kuckartz [28], which allows categories to emerge from the data itself, following five iterative steps:

1. Defining the purpose of category formation – determining what research questions the categories should address and considering prior knowledge.
2. Determining category type and level of abstraction – deciding whether categories are factual (e.g., professions), thematic (e.g., climate change), evaluative (e.g., intensity of an emotion), or analytical (derived through deeper data interpretation).
3. Familiarizing with the data and defining coding units – reviewing all responses and deciding whether to segment data into independent, self-contained meaning units.
4. Iterative coding process – reading through responses sequentially, assigning existing categories or forming new ones, and refining the system as needed.
5. Systematizing and structuring categories – organizing similar categories into main and subcategories, ensuring a clear and manageable coding system, and counting frequencies of mentioning across participants per (sub-)category.

Through this process, common themes and needs regarding the design of crisis/ retreat rooms were identified.

## Results

### Sample

Thirty participants were recruited (*n* = 14 female, *n* = 16 male). Random assignment to furniture form was unbalanced with *n* = 12 to curved and *n* = 18 to angular. Mean age was 40.3 years (*SD* = 13.5, range 22–74 years). Approximately 60% of participants had a prior ICD-10 F2 diagnosis (schizophrenia spectrum or delusional disorders), and 30% had an F3 diagnosis (affective disorders, mostly bipolar disorder). One participant had been diagnosed with F1 (substance use), one with F4 (PTSD), and one had missing diagnosis data. Concerning frequency of past isolation, participants reported two on average (*M* = 2.2, *SD* = 2.8, range 1–15), with most individuals (80%; *n* = 24) knowing our ward’s seclusion rooms by own experience. Since exact dates of the last seclusion experience were often unknown, we compared the year of the experiment with the reported year of the most recent seclusion. Four participants had missing data. For most (*n* = 19; 63%), the last isolation experience occurred in the same year or the year prior to study participation. In five cases, 3–8 years had passed, in four cases, the time elapsed exceeded ten years. Despite this variation, all participants confirmed that they remembered the isolation experience and room clearly. Two participants reported not having a formal school degree (~ 7%), two reported having the lowest possible one in Germany (~ 7%), four a medium (~ 13%), and the majority (*n* = 14, 57%) the highest attainable German school degree (57%). Five participants had missing data on education.

### Overall effects of wall design, furniture form, and their interaction on participant ratings

A strong main effect of (within-subject) room design was found for all four dependent variables. Wall design significantly influenced ratings of restfulness (Wilks’ *Λ* = 0.272, *F*(9, 20) = 5.961, *p* < .001, *η²*_partial_ = 0.728), stress (Wilks’ *Λ* = 0.236, *F*(9, 20) = 7.201, *p* < .001, *η²*_*partial*_ = 0.764), liking (Wilks’ *Λ* = 0.103, *F*(9, 20) = 19.296, *p* < .001, *η²*_*partial*_ = 0.897), and overwhelm (Wilks’ *Λ* = 0.412, *F*(9, 19) = 3.009, *p* = .021, *η²*_*partial*_ = 0.588).

The exploratory between-subjects effect of furniture form (angular vs. curved) was not significant for any variable, with restfulness (*F*(1, 28) = 0.047, *p* = .829, *η²*_*partial*_ = 0.002), stress (*F*(1, 28) = 1.451, *p* = .239, *η²*_*partial*_ = 0.049), liking (*F*(1, 28) = 0.035, *p* = .853, *η²*_*partial*_ = 0.001), and overwhelm (*F*(1, 27) = 0.382, *p* = .542, *η²*_*partial*_ = 0.014) all showing no significant differences based on furniture shape.

Exploratory interaction effects between wall design and furniture form did not reach statistical significance for any of the variables: restfulness (Wilks’ *Λ* = 0.855, *F*(9, 20) = 0.378, *p* = .932, *η²*_*partial*_ = 0.145), stress (Wilks’ *Λ* = 0.727, *F*(9, 20) = 0.834, *p* = .594, *η²*_*partial*_ = 0.273), liking (Wilks’ *Λ* = 0.674, *F*(9, 20) = 1.075, *p* = .422, *η²*_*partial*_ = 0.326), and overwhelm (Wilks’ *Λ* = 0.705, *F*(9, 19) = 0.884, *p* = .556, *η²*_*partial*_ = 0.295).

In the following, the significant main effect of wall design will be followed up with post-hoc tests. Further statistical details for the post-hoc tests can be found in the Supplementary Materials B Excel file.

### Effects of individual wall designs on participant ratings of restfulness, stress, liking, and overwhelm

**Fig. 2:**
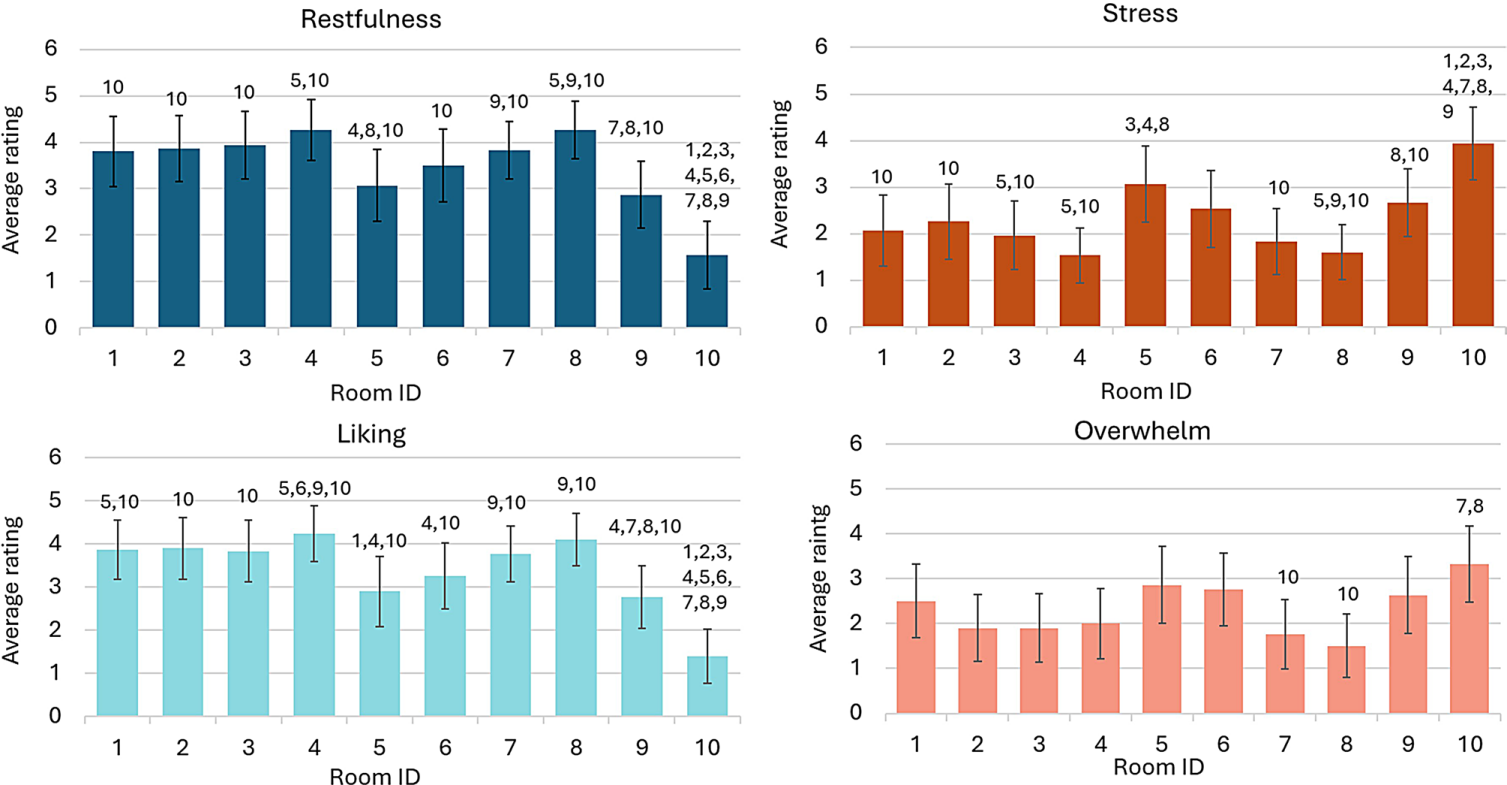
Average participant ratings per room design (1–10) across the four rating dimensions. Note. Significant differences between rooms are indicated by small numbers (indicating a significant difference to the respective room design (1–10) above the respective bars). Error bars represent 95% (2*SE) confidence intervals around the respective means. (1) Pine Tree Forest, (2) Königsstuhl (Chalk Cliff on the Baltic Sea Island ‘Rügen’), (3) Oak Tree on a Meadow, (4) Dunes at Baltic Sea, (5) River Running Through Wilderness, (6) Grass Field Near Mountain With Waterfall, (7) green wall, (8) blue wall, (9) beige wall, (10) neutral (white, empty) control room

Figure 2 depicts mean ratings, 95% confidence intervals, and significant differences found across rating dimensions and room designs.

#### **Restfulness**

All designed rooms (Rooms 1–9) were rated as significantly more restorative than the empty control room (all *p*_*FDR*_ < 0.02). Among the designed rooms, the *beige* wall design room was rated as significantly less restorative than both the *blue* (*p*_*FDR*_ = 0.014) and the *green* (*p*_*FDR*_ = 0.029) wall design rooms. In addition, the motif *River Running Through Wilderness* was perceived as significantly less restorative than both the *Dunes at Baltic Sea* motif (*p*_*FDR*_ = 0.046) and the *blue* wall (*p*_*FDR*_ < 0.05).

#### **Stress**

All designed rooms were rated as significantly less stressful than the control room (all *p*_*FDR*_ < 0.04). Among the designed rooms, the *beige* wall was perceived as significantly more stressful than the *blue* wall (*p*_*FDR*_ = 0.032). Additionally, motif *River Running Through Wilderness* was rated as significantly more stressful than the motifs *Dunes at Baltic Sea* (*p*_*FDR*_ = 0.013), *Oak Tree on a Meadow* (*p*_*FDR*_ = 0.040), and the *blue* wall design (*p*_*FDR*_ = 0.036).

#### **Liking**

All designed rooms were rated as significantly more liked than the control room (all *p*_*FDR*_ < 0.01). Among the designed rooms, the *beige* wall design room was liked significantly less than the *blue* wall design room (*p*_*FDR*_ = 0.007), the motif *Dunes at Baltic Sea* (Room 4; *p*_*FDR*_ = 0.039), and *green* wall design room (*p*_*FDR*_ = 0.036). Additionally, motif *River Running Through Wilderness* was liked significantly less than *Dunes at Baltic Sea* (*p*_*FDR*_ = 0.039) and *Pine Tree Forest* (*p*_*FDR*_ = 0.038).

#### **Overwhelm**

The control room was perceived as significantly more disturbing than both the *blue* wall (*p*_*FDR*_ = 0.019) and *green* wall (*p*_*FDR*_ = 0.031) designs but did not differ from any other designed rooms.

#### **Card sorting (overall ranking)**

The ranking task revealed a clear overall preference gradient across the designs based on the average assigned ranks per design. All designed rooms (Rooms 1–9) were ranked significantly better than the white empty control room (all *p*_*FDR*_ < 0.01). Post-hoc tests confirmed that the *Dunes at Baltic Sea*, *blue*, and *green* wall designs were consistently and significantly preferred over both the *beige* and the *River Running Through Wilderness* wall motif (all *p*_*FDR*_ ≤ 0.02). While some other nature motifs, namely the *Königsstuhl (Chalk Cliff on the Baltic Sea Island ‘Rügen’)*, *Oak Tree on a Meadow*, and *Grass Field near Mountain With Waterfall*, were also ranked significantly higher than the *beige* wall, none of them was simultaneously ranked higher than any other wall design. For details, please refer to the Supplementary Materials Excel file.

### Qualitative results

The content analysis of participant responses (*N* = 30) resulted in 221 codes in five key thematic categories, with 17 subcategories related to experiences and needs regarding crisis rooms. These categories emerged through systematic coding and analysis of participant statements and are reported with frequency counts (n), key topics and if insightful an anchor example of a participant reference (*IDs*) (Table 3). Individuals with lived experience emphasized the need for rooms resembling normal living environments, with comfortable furniture, beds, calming colors such as blue and green, and nature motifs. Adjustable lighting, operable windows with outside views, and orientation aids (e.g., clocks, written information) were seen as essential, while either sterile or overstimulating designs were rejected. Continuous access to staff and visitors was regarded as crucial, alongside transparent communication and respectful treatment. Coercive measures were strongly criticized, with calls to avoid visible reminders such as straps or cameras. Participants further highlighted the value of recreational opportunities, including books, media devices, and options for physical activity. Finally, hygiene was a recurring theme, with demands for cleaner spaces, functional sanitary facilities, and personal hygiene products.

**Table 3:** Qualitative analysis of needs and preferences of individuals with lived experience in psychiatric crisis rooms

**Categories (N)**	
Subcategories (n)	Topics*/Key Codes (ID)*
**Design** (117)	
Room design (17)	need for simple, calm, soothing environment, soft features, not too many stimuli, assemblance to a ‘normal room’, calming auditory stimuli (e.g., nature sounds), aromatherapy, fresh air, sufficient room size
Wall design (28)	colouring/ wall visuals preferred to blank walls: warm/ soft colours, particularly blue and green, nature motifs: especially ocean, water, or dunes, avoid overwhelming details or intricate visuals, not covering the entire wall / *allowing patients to choose where to rest their gaze (ID 06)*
Light (13)	importance of adjustable lighting/jalousies, warm and decent light / l*ights must not be on all night - it is best to decide for yourself (ID 08)*
Doors, windows (14)	being able to open the windows and doors themselves, view outside, fresh air, ventilation, open door
Furnishing/décor (33)	furnished room with décor features: covers, cushions, carpets, real plants, need for a comfortable bed with bedclothes and cover; opportunity to sit: chairs, sofa, table, closet
Orientation (12)	basic temporal awareness, spatial orientation: clocks, access to windows; need for transparent, early information about the situation, written documentation about their diagnosis, treatment status; scheduled staff visits, names and pictures of staff, specifically responsible physician / *openness of information: explain the patient what is currently happening and under what conditions they can leave again*,* as well as the truth about their situation (ID 27)*
**Contact and Communication (26)**	
General contact (12)	Visitors allowed, regular contact, human interaction / *keep in touch with the outside world (ID 02)*
staff contact (6)	consistent staff presence / *staff needs to be there*,* especially during fixation (ID 10*)
contact access (7)	reliable response system / *staff should be there or quick to call; call button (ID 06)*
**Therapeutic Approach (28)**	
Handling/ understanding needs (8)	transparency, staff qualification, therapeutic support, debriefing, patients’ individual needs, respectful treatment / *be treated on eye-level (ID 25)*
Restrictive/coercive measures (14)	concerns about the use of coercive measures, advocating for humane approaches, open-door policies, alternatives to strong sedation, no visual reminders of former violence/ coercive measures triggering negative emotions, hide fixation straps, grids and cameras indicating a threatening environment / *use of physical restraint shouldn’t be as it is*,* it should be an exception (ID 07) / traces of violence are burdensome and scary*,* I get even more panic (ID 09)*
Basic care (9)	need for food and drinks during isolation, access to running water, toilet flush
**Recreational Activity (31)**	
Recreational Opportunities (9)	importance of safe recreational options: books, arts supplies, fixed installations
Technical devices (12)	listen to music, podcasts, radio, access to mobile phones TV / *human voices (ID 11)*
Physical activity (10)	physical exercise equipment, meditation applications, yoga gear, punching bags
**Hygiene (19)**	
Hygiene standards (9)	poor existing hygiene standards, importance of a clean, hygienic environment, regularly clean the room, use of disinfection; availability of personal sanitary products: toothbrush, paste, soap, toilet paper / *Cleanliness is super important! (ID14)*
Sanitary arrangements (10)	usable sanitary facilities with a door, flushable toilet, non-steel, toilet seat

## Discussion

The present study focused on pre-testing different wall-design options for seclusion rooms to inform a redesign initiative within a psychiatric acute (emergency) setting, involving *N* = 30 individuals with lived experience of psychiatric coercive isolation/seclusion in such rooms. Our findings contribute to the emerging evidence that the use of nature wall imagery can meaningfully affect patients’ psychological states in healthcare settings. Systematic research on this topic in psychiatric settings is highly rare, with prior research mostly focusing on waiting areas or procedure rooms in non-psychiatric healthcare settings [20]. By systematically comparing responses to different renders of the redesigned rooms with different wall colors and photographic nature motifs, we found that nature-themed designs, particularly blue and green hues and a calm natural scene (*Dunes at Baltic Sea*), were consistently rated as more restorative and likable, as well as less stressful. These designs were also explicitly mentioned as desirable in qualitative participant accounts. By contrast, an empty white control room and, to a lesser extent, a beige-painted room were perceived as less calming or likable and more stressful, suggesting that ‘neutral’ colors or deprived environments may not support emotional regulation in an acute psychiatric crisis room. Our findings are consistent with guideline recommendations and prior evidence that advocate the inclusion of soothing colors and natural imagery in healthcare design in general, including acute psychiatric settings [8, 9, 11], providing targeted evidence for more beneficial user responses to specific wall designs in a specific setting (seclusion rooms within an acute psychiatric ward).

Importantly, this study was not only about evaluating specific designs but also about co-developing a multi-method approach for shared decision-making with individuals with lived experience of coercive isolation within an acute psychiatric treatment. By engaging patient boards and individuals with lived experience both through structured ratings and open questions, we implemented a feasible approach to collaboratively and systematically assess preferences and needs for this specific setting, which may be useful for usage in future studies. Albeit co-design studies, including in acute psychiatry, have been conducted before (e.g [25]), our study illustrates how individual design factors could be investigated in a targeted manner, alongside open inquiry in users about their preferences.

Furthermore, results hint towards the need to adapt wall motifs to specific contexts and user groups. Notably, an image depicting a river running through a rocky wilderness was rated as less restorative and more stressful than other nature-based scenes. Prior research has shown that lower visual coherence, legibility, and accessibility are associated with increased danger appraisals, such as in dense forest environments [29]; properties that also apply to this specific image. Likewise, a photograph of a chalk cliff on a Baltic Sea island (‘Königsstuhl’, Rügen, Germany) may have elicited fear of height, associations with danger, or even suicidal connotations. These nuances indicate that not merely the presence of natural imagery, but scene composition, emotional tone, and viewer characteristics (e.g., sensation-seeking or sex [30]) influence responses to nature motifs. In a coercive isolation room, where patients have little choice to avert their gaze and experience substantial emotional burden, careful selection of visual stimuli is therefore crucial.

Importantly, the vast majority of previous studies documenting benefits of nature imagery were conducted in different contexts than the present one, such as waiting rooms, procedure rooms, or somatic inpatient wards, where patients retain greater agency over where to look, are not confined, and likely experience considerably lower mental distress [20]. Coercive seclusion, by contrast, constitutes an extreme perceptual and emotional context in which visual stimuli are unavoidable and may, under certain circumstances, amplify rather than alleviate stress. Existing architectural guidelines for acute psychiatry do not provide concrete recommendations for specific wall designs (color or motif), but instead broadly emphasize soothing, naturalistic, and non-institutional materials [7–9]. Our results provide evidence that calm, natural wall designs are helpful, yet further in-vivo research is needed to assess how such designs function under the real-world psychological load of involuntary isolation. However, a prior study by Faerden et al. [25] was conducted in a secluded ward of a psychiatric unit, comparable to the present study’s context, and it was found that a redesign targeted at enhancing dignity that (however among many other elements) also included nature wall images, resulted in staff perceiving the environment as supportive for both caring for patients and patient outcomes. This is broadly in line with what we quantitatively found concerning wall imagery and qualitatively concerning the needs of individuals with lived experience.

In terms of furniture form (angular vs. curved), tested on a purely exploratory basis and subtle nature of manipulations, our data provides no evidence that this factor significantly affected psychological responses, despite prior research suggesting that curved designs can elicit more positive affective and aesthetic reactions [31, 32], including for 2D stimuli of interior design (furniture) [32]. These studies were all conducted in healthy participants and to our knowledge no targeted experimental studies exist in psychiatric patients. Null results in the present study most likely reflect the limited salience of the form manipulation in our projected settings and/or low power due to the small sample size. To clarify how these design effects operate in psychiatric contexts and samples, experimental research with more pronounced form manipulations and larger samples is needed. In addition, it may be advisable to separate studied target experimental factors (wall design, furniture design) or test their interaction in a systematic approach.

Open participant responses reiterate that individuals in confined psychiatric spaces desire designs and related social interactions that emphasize dignity, comfort, and maintaining autonomy and control; needs that are rarely met during coercive isolation in conventional psychiatric seclusion rooms. Design-wise, individuals with lived experience emphasized the importance of light, fresh air, cleanliness, possibility to use the rooms as open retreat spaces, and the ability to orient themselves in time and space. Several study participants expressed that a well-designed room might have helped them to regulate their emotions better. These demands are supported both by existing guidelines for isolation rooms [8, 9] and research that identified similar factors as crucial in patient experiences of coercion, including isolation [33, 34]. Coercive measures, such as coercive confinement, are a last resort, and patient needs as reported above must be addressed to reduce stigma and trauma, and to support stabilization.

### Limitations

The sample size was small (*N* = 30) and skewed towards individuals with higher education. The timing of the last isolation experiences in a psychiatric seclusion room varied considerably, albeit most participants had experienced this last during the past one to two years and reported vivid memories in general. Additionally, the stimuli focused primarily on wall design (visual sense), whilst other relevant senses and features such as smell, lighting, brightness, sound, temperature or interactive aspects were omitted. This reductionist approach was necessary to study specific effects and to ensure cognitive accessibility and feasibility for our participant population, but ecological validity is consequentially limited. This could be achieved in future studies using (multimodal) Virtual Reality (VR) paradigms, specifically applying to the manipulation of form, which may be experienced more strongly in a 3D space. Furthermore, while the study was developed with input from individuals with lived experience, including co-design sessions on questionnaire content, rating formats, and general room elements—we acknowledge that not all design decisions were made in a participatory manner. In particular, the selection of wall imagery was conducted by the research team and affiliated colleagues from the fields of (environmental neuroscience) psychology, (landscape) architecture, and clinical care. This decision was driven by the need to vary landscape types and properties to obtain a higher possible divergence in ratings. Another limitation concerns the items that were used for the ratings of room designs. Although the selected single-item measures to assess restfulness/restoration, stress, and liking were theory-informed, our emphasis was on practicality, with items being tailored for the current study in consultation with clinicians and individuals with lived experience. Hence, comparability with previous studies remains limited, and external validity will need to be further established in future work. In addition to subjective ratings, it would be advisable to assess objective responses to the designs, such as physiological arousal (e.g., electrodermal activity, heart rate, etc.) or eye movements (e.g., measured via eye tracking). Concerning the qualitative study part, it can be criticized that no in-depth interviews were conducted, and verbatim content was not recorded (e.g., using audio equipment), while relying on notes (albeit those were validated/confirmed by participants). This approach was adopted to assure patient trust, an acceptable time frame (1.0–1.5 h), and minimal participant burden. Last, current levels of patient distress or symptom severity were not systematically assessed. While experimenters perceived participants as sufficiently reliable and stable enough for participation, formal clinical assessments could strengthen future studies.

## Conclusion

The present findings provide preliminary evidence that individuals with lived experience of coercive isolation in psychiatric seclusion rooms respond more favorably to suggested room (re-)designs with calming natural, cool-toned wall color schemes. Participants preferred layouts featuring blue and green walls and a photorealistic, non-complex nature wall motif (grass-covered dune by the sea). In contrast, more neutral wall designs (beige color), a rougher wilderness scene, and a sensory-deprivation environment (empty white seclusion room) elicited less favorable responses, suggesting that wall design should balance under- vs. overstimulation. Qualitative accounts further emphasized the importance of maintaining dignity, autonomy and environmental control, while offering comforting and nature-inspired design elements, action possibilities, positive human contact, and orientation, highlighting that design choices can support emotional safety and a more humane experience.

## Supplementary Information

Below is the link to the electronic supplementary material.

Supplementary Material 1

Supplementary Material 2

## Data Availability

All relevant data and materials are available from the first author upon reasonable request.

## Declarations

### Author statement

All authors agreed to the submission and publication of this manuscript. Data is available from the first author upon reasonable request.
