# Supplementary material for: Informing the redesign of psychiatric seclusion rooms: a mixed-methods pre-evaluation with individuals with lived experience
Source: BMC Psychiatry. 2026 Jan 16;26:58. doi: 10.1186/s12888-026-07780-0 (PMC12828918; doi:10.1186/s12888-026-07780-0)
Supplement: Supplementary file 1 — Supplementary Material 1 [file 12888_2026_7780_MOESM1_ESM.docx]

**Supplementary Materials**

**Key excerpts from the questionnaire (translated from German)**

The following 5 items were assessed for each of the ten room designs, which were presented in random order (see main paper for details). The rating format was discussed and refined with the local scientific advisory board of individuals with lived experience (EmPeeRie) of the University Medical Center Hamburg-Eppendorf, Clinic and Policlinic for Psychiatry and Psychotherapy.

**Imagine you are in a state of psychological crisis. You are in the room shown...**

| **1** | What goes through your mind or what do you feel when you see this room? Ideally, name 3 describing words (adjectives) (or just freely associate) |
| --- | --- |
| **2** | How restorative do you find the room overall?  Little/ not restorative  Very restorative |
| **3** | How stressed do you feel by the room overall?  Not stressed at all  Very stressed |
| **4** | How much do you like the room overall?  Don’t like it at all  Like it very much |
| **5** | Does the wall design unsettle, burden, or overwhelm you?  Not at all unsettling/ overwhelming  Very unsettling/ overwhelming |

**Your task:
Please take the deck of room cards and sort the rooms from left to right.
Which room would you prefer if you were in a psychological crisis?
Which one would come second, third, … and which one would be your least preferred?**

**@ Experimenter:**Please record the order (enter room codes in the corresponding positions).

| 1  HIGHEST PREFERENCE | 2 | 3 | 4 | 5 | 6 | 7 | 8 | 9 | 10  LOWEST PREFERENCE |
| --- | --- | --- | --- | --- | --- | --- | --- | --- | --- |
|  |  |  |  |  |  |  |  |  |  |

On the following scale, how well were you able to imagine yourself in the situation in the room?

Please mark a point on the line

**0%** I----------------------------------------------------------------------------------------------------I **100%**

***Could not imagine it at all***  ***Could imagine it very well***

**In your opinion, what should an ideal 'crisis room' or retreat space in a psychiatric ward look like/ offer to help you calm down during a psychological crisis?**
What would you need or wish for in such a space?
What might disturb or unsettle you?
What should absolutely be avoided?

**@Experimenter:** Please note down key points; mention **open/ voluntary retreat spaces** to avoid propagating seclusion. Discuss notes with patient to confirm validity.

__________________________________________________________________________________

____________________________________________________________________________________________________________________________________________________________________________________________________________________________________________________________________________________________________________________________________________________________________________________________________________________________________________________________________________________________________________

*Not reported as part of the main paper and analyses, but formally assessed:*

**Rate the participant’s response reliability on a scale from 1 to 5, using the following criteria:**

**1 = Low reliability**
Unclear whether the questions or response format (scale) were understood. A high degree of prompting, structuring questions, or explicit suggestions is required to elicit any response at all. Some items may remain unanswered.

**2 = Rather low reliability**

**3 = Moderate reliability**
Questions were understood independently. The response format (scale) was understood or understood with some support. The traffic light aid may have been used. Occasional clarification or examples (e.g., suggesting adjectives like “fast”) were needed. The participant was generally able to communicate clearly without leading questions or suggestions.

**4 = Rather high reliability**

**5 = High reliability**
The participant clearly understood both the questionnaire and the response format (scale), and was able to express themselves clearly and consistently.

**Reliability: __________________**

*We decided not to exclude any participants based on perceived response reliability, as even seemingly incoherent or inconsistent answers (from our perspective) were considered valuable and reflective of genuine lived experience.

In addition, we collected patient responses regarding the potential implementation of a media wall within the rooms. This included preferences for specific uses, as well as perceived benefits and risks. These data are **not part of the current article or supplement**, but are available upon request from the first author: [l.ascone-michelis@uke.de](mailto:l.ascone-michelis@uke.de)

**Wall motif selection process**

To ensure a systematic and theoretically grounded selection of nature-based wall images, a structured pre-study was conducted prior to the main study. The goal was to identify suitable nature scenes for simulated wall projections in psychiatric crisis rooms, based on their potential restorativeness, emotional tone, and perceived safety.

Twenty high-resolution landscape photographs were selected and categorized into five established visual landscape types: *canopied*, *enclosed*, *feature*, *focal*, and *panoramic* (cf. Litton, 1968). Each category included two images with and two without visible water elements, resulting in a visually and thematically balanced stimulus set (N = 20). Images were screened for technical and psychological suitability (e.g., realistic daylight conditions, absence of people, animals, or buildings, no extreme contrast editing, no directly threatening elements, realistic human/ no bird-eye perspective, high resolution, good weather/ clear weather conditions).

A panel of seven experts—drawn from fields such as landscape architecture, environmental psychology, psychotherapy, geography, and architecture—rated each image on a comprehensive custom online questionnaire comprising the following evaluative assessments:

- Affective dimensions (adapted from Stemmler et al., 2001)
  - Valence
  - Arousal
  - Fear
- Perceived Restorativeness Scale (PRS; Hartig et al., 1997; Kaplan, 1995)
- Contemplative Landscape Model (CLM; Olszewska et al., 2016)

The order was partially randomized to reduce order effects. All ratings were collected via visual analogue scales (0–100) (i.e., PRS and CLM were customized accordingly).

To systematically identify the most suitable wall images, we calculated standardized mean scores (z-scores) per image and per dimension. Rankings were assigned within each image category (*canopied*, *focal*, etc.) and across all images, following these steps:

1. **Dimension-level ranking within categories**: For each rated dimension (valence, arousal, fear, PRS, CLM), images received a rank from 1 to 4 within their landscape category (e.g., canopied), where **4 = highest rank** on a given dimension.
2. **Dimension-level ranking across categories:** For each rated dimension (valence, arousal, fear, PRS, CLM), images received a rank from 1 to 20 across all landscape categories, where **20 = highest rank** on a given dimension.
3. **Summed rank calculation within categories**: For each image, we summed its category-level ranks across the five dimensions. This yielded a **total rank score** (maximum = 20), representing its composite suitability based on affective and theoretical evaluation. The 7 images with the 5 highest summed ranks (some images had the same summed rank) were selected for further discussion.
4. **Summed rank calculation across categories**: For each image, we summed its across-category ranks across the five dimensions. This yielded a **total rank score** (maximum = 100). The 5 images with the 5 highest summed ranks were selected for further discussion.
5. **Comparison and discussion:** Based on the scores calculated in step 3 and 4, the images with the highest ranks within and across categories were compared. Differences between rankings on separate dimensions as well as contraindications for certain images were discussed.
6. **Final selection**: The five images with the highest total rank scores—across and within a diverse set of landscape categories—were selected for use in the main study. One additional image was added to ensure variety between images and image ratings, resulting in a total of six images.

While the procedure equally weighted all five dimensions, this was a deliberate decision to balance affective (valence, arousal, fear) and theoretical (PRS, CLM) considerations. As the goal was not hypothesis testing but diversity-aware stimulus selection, we prioritized variety and richness of potential user reactions while simultaneously ensuring that images with very low ratings on all dimensions were not included.

We additionally collected ratings on several more affective dimensions (e.g., sadness, serenity, complexity). However, due to concerns over cognitive load, psychometric overlap, and interpretive clarity, we decided to focus on a core set of five evaluative dimensions. The full set of data remains available for future analysis or replication efforts.

Further details and data from the ranking procedure, including the full stimulus set are available from the first author upon request.

**References**

Hartig, T., Kaiser, F., & Bowler, P. (1997). *Further development of a measure of perceived environmental restorativeness* (Working Paper #5). Institute for Housing Research, Uppsala University. https://uu.diva-portal.org/smash/get/diva2:130237/FULLTEXT01.pdf

Kaplan, S. (1995). The restorative benefits of nature: Toward an integrative framework. *Journal of Environmental Psychology*, *15*(3), 169–182. https://doi.org/10.1016/0272-4944(95)90001-2

Litton, R. B., Jr. (1968). *Forest Landscape Description and Inventories: A Basis for Land Planning and Design*. Research Paper PSW-49). U.S. Department of Agriculture, Forest Service, Pacific Southwest Forest and Range Experiment Station.

Olszewska, A. A., Marques, P. F., Ryan, R. L., & Barbosa, F. (2016). What makes a landscape contemplative? *Environment and Planning B: Urban Analytics and City Science*, *45*(1), 7–25. https://doi.org/10.1177/0265813516660716

Stemmler, G., Heldmann, M., Pauls, C. A., & Scherer, T. (2001). Constraints for emotion specificity in fear and anger: The context counts. *Psychophysiology*, *38*, 275–291.
